# Supplementary material for: Systemic and vascular inflammation in an in-vitro model of central obesity
Source: PLoS One. 2018 Feb 13;13(2):e0192824. doi: 10.1371/journal.pone.0192824 (PMC5811040; doi:10.1371/journal.pone.0192824)
Supplement: S1 File — (PDF) [file pone.0192824.s001.pdf]

Systemic and vascular inflammation in an in-vitro model of central obesity

Arti Ahluwalia, Alessandra Misto, Federico Vozzi, Chiara Magliaro, Giorgio Mattei, Maria Cristina Marescotti, Angelo Avogaro, Elisabetta Iori

SUPPORTING INFORMATION

Table A reports the change in metabolite values for 1-way HUVEC and 1-way Hepatocytes for completeness (the main text only reports the 1, 2 and 3-way adipose tissue data, given that the analysis of metabolite changes due to changes in adiposity was the main objective of the study).

Δ Metabolite	HepG2	HUVEC
TRG (mM)	0.038 ± 0.009	0.013 ± 0.016
Glycerol (mM)	0.014 ± 0.008	0.044 ± 0.016
FFA (mM)	0.130 ± 0.018	0.049 ± 0.017
HA (μg/mL)	3.380 ± 1.128	0
Glucose (mM)	-0.800 ± 1.280	1.980 ± 0.510
Lactate (mM)	0.145 ± 0.290	1.750 ± 0.170
Urea (μg/mL)	18.710 ± 0.270	44.400 ± 1.230

Table A. Change in metabolite values for 1-way HUVEC and 1-way Hepatocytes

Figures A, B and C show the data for all metabolites and markers at 4 and 24 hours. In the main text we focused on the 24 hour data in order to highlight changes as well as for the sake of comparison with our earlier study. In general, the metabolites were more stable than the pro-inflammatory markers, which tended to increase from 4 to 24 hours. Figure D reports the whole dataset showing changes in IL-6 and MCP-1 levels at 24 h for all the 11 conditions (1-way HUVEC, 1-way Hepatocytes and the 9 AT dependent 1-, 2- and 3-way conditions. The Figure also shows the 2 way ANOVA analysis for MCP-1 and IL-6 focusing on the way effect (the AT affect is reported in the main text).

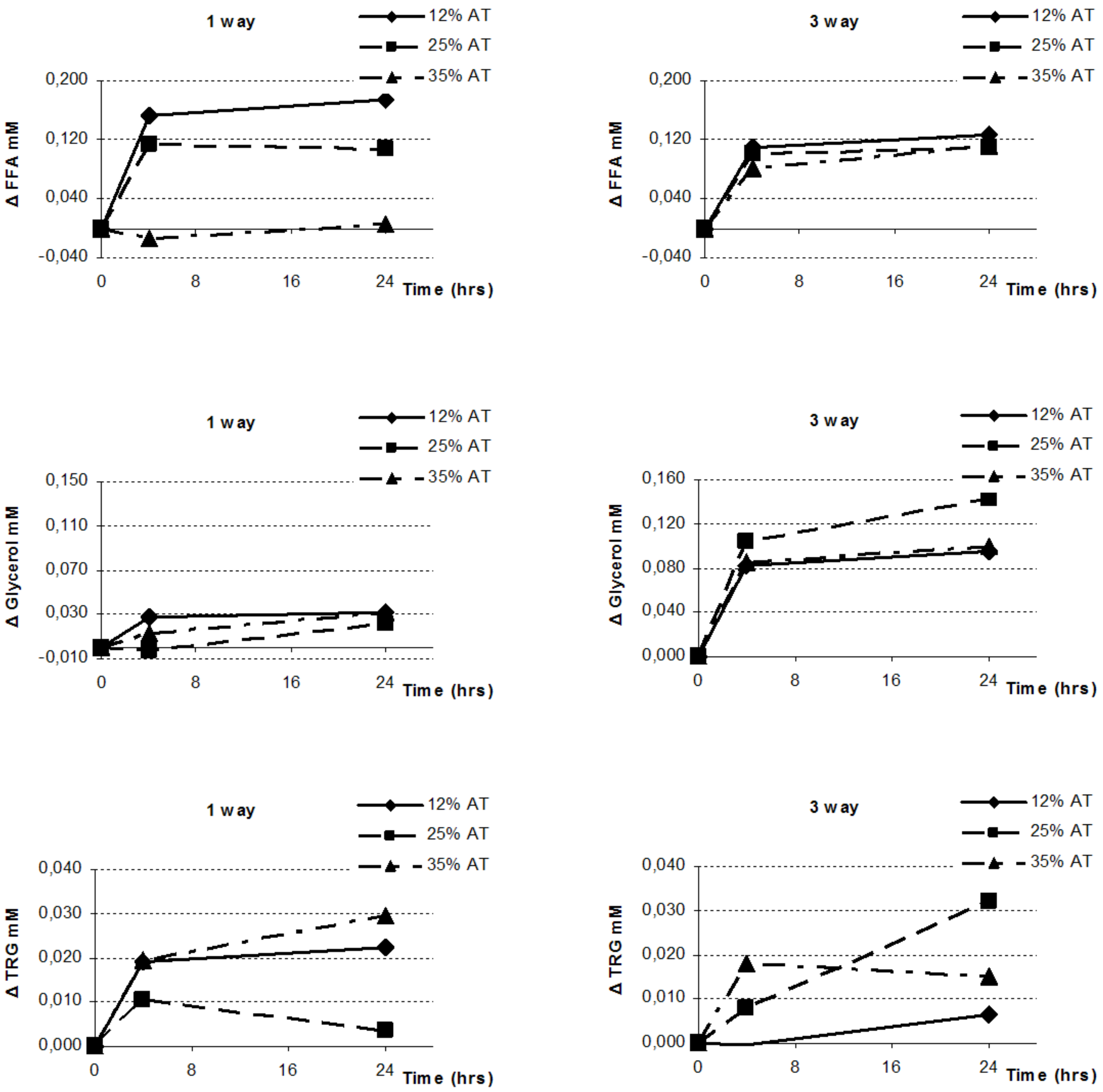

Fig. A. Changes in FFA, glycerol, and triglyceride concentrations in the 1 and 3-way cultures with different amounts of adipose tissue and at different time points (4 and 24h). Metabolite medium concentrations at time 0 were subtracted from to the medium concentrations at various time points.

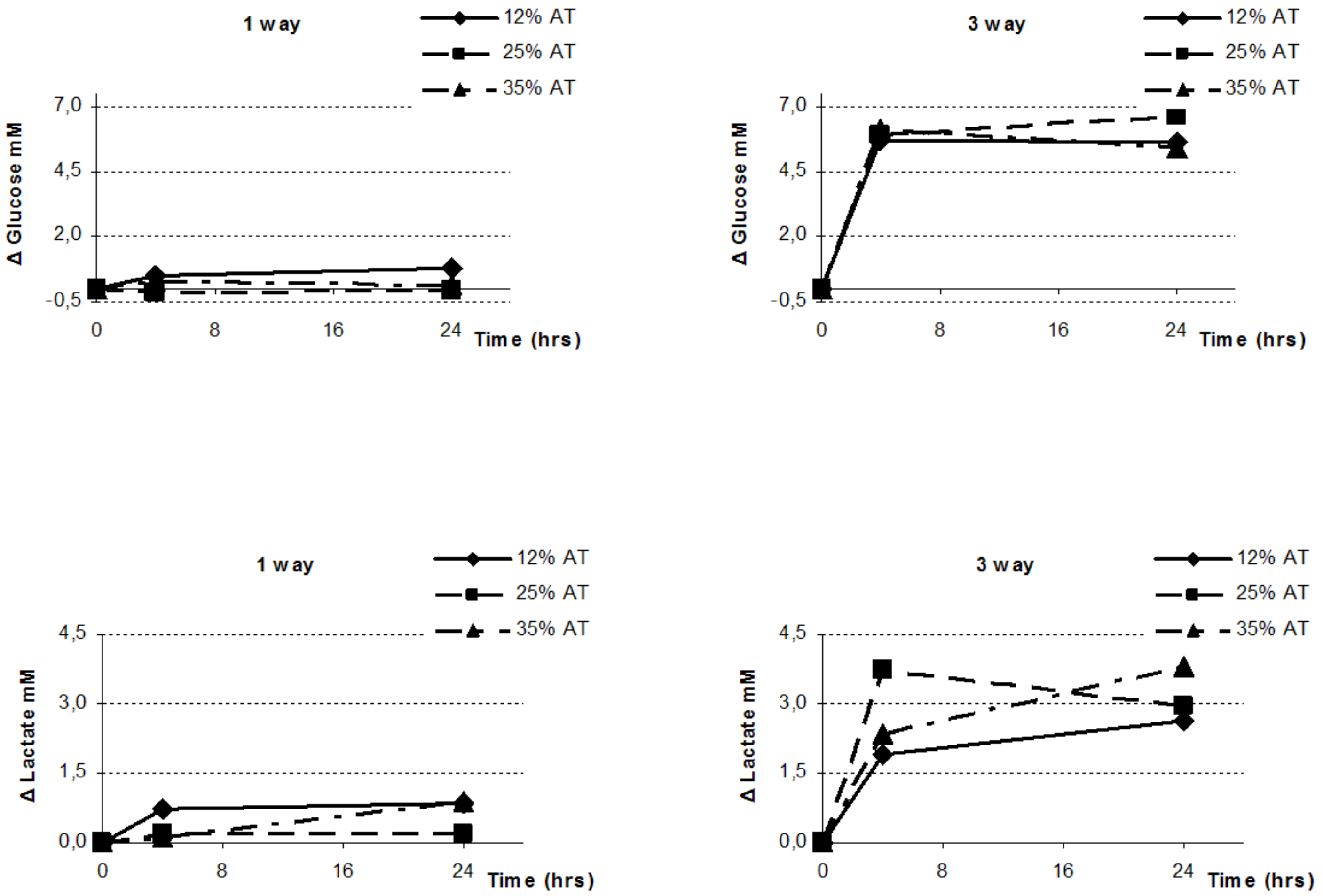

Fig. B. Changes in glucose and lactate concentrations in the 1 and 3-way cultures with different amounts of adipose tissue and at different time points (4 and 24h). Metabolite medium concentrations at time 0 were subtracted from to the medium concentrations at various time points.

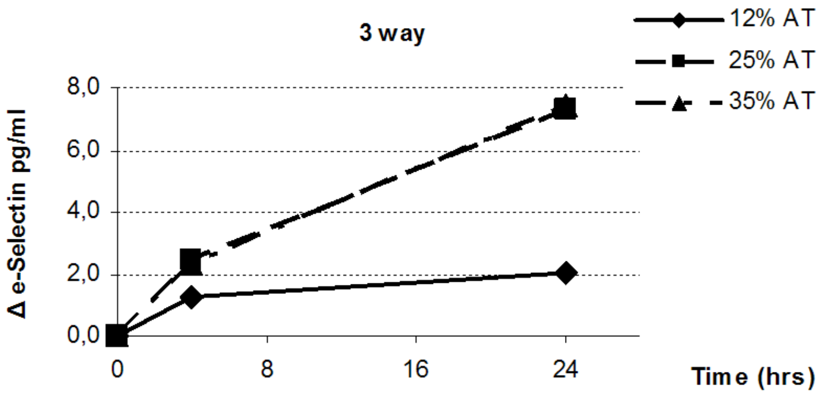

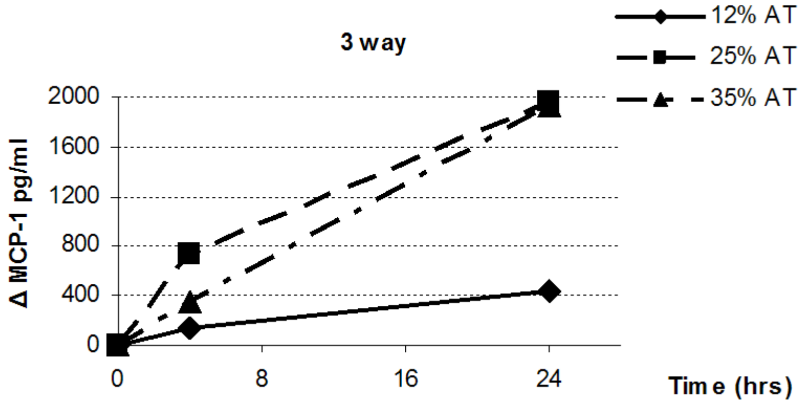

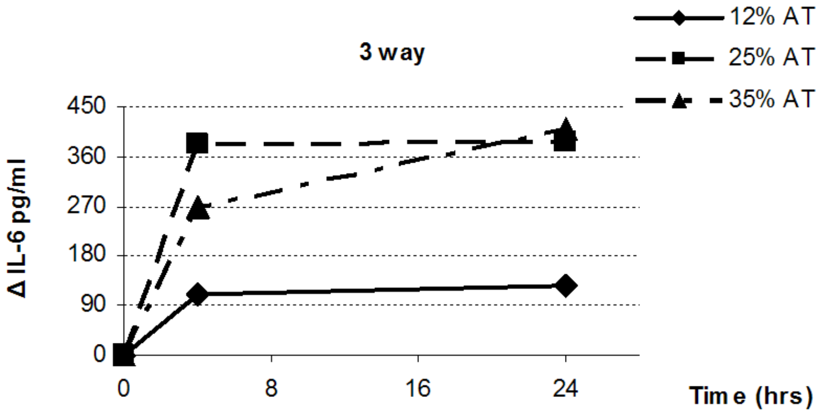

Fig. C. Changes in IL-6, MCP-1 and e-selectin concentrations in the 3-way cultures with different amounts of adipose tissue and at different time points (4 and 24h). The concentrations at time 0 were subtracted from the medium concentrations at various time points.

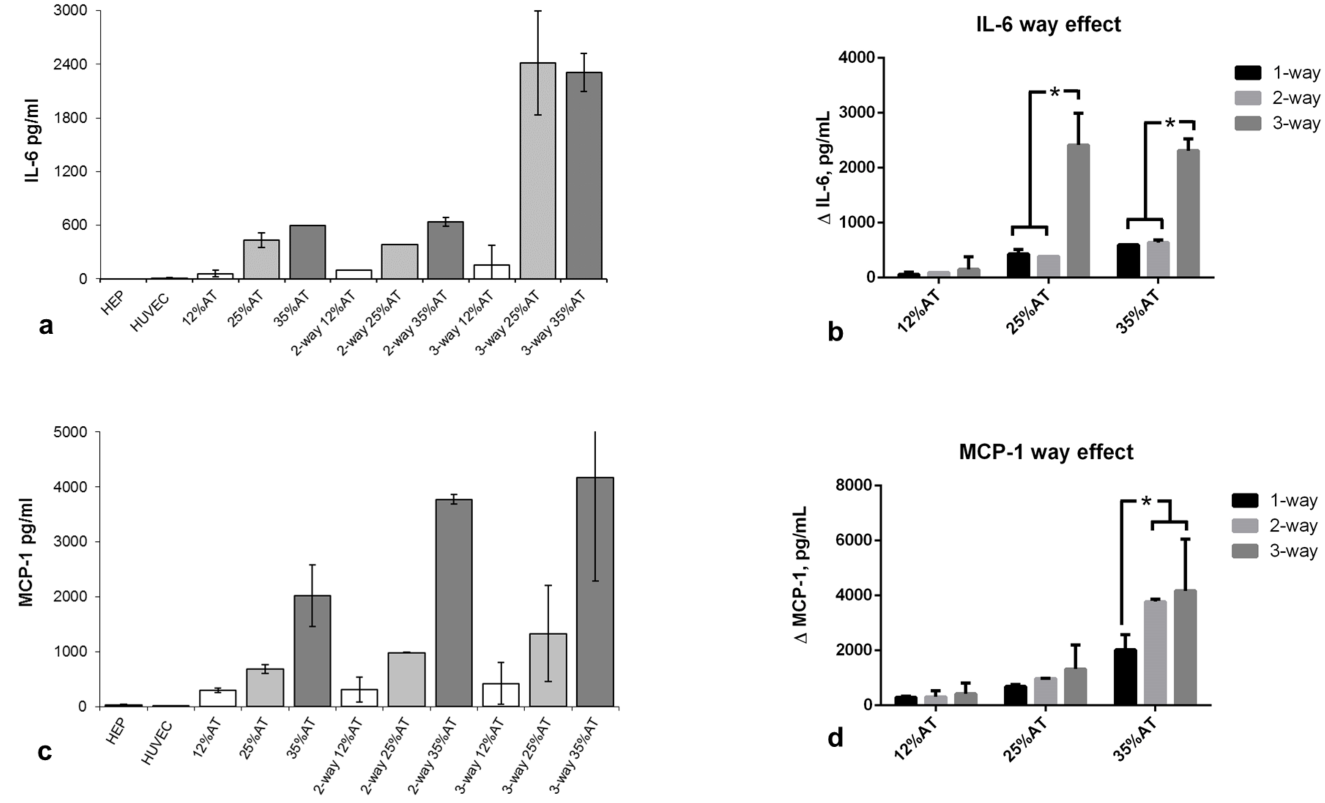

Fig. D. IL-6 and MCP-1 a) IL-6 concentrations at 24h in all conditions; b) 2-way ANOVA analysis for IL-6 showing the way-effect; c) MCP-1 concentrations at 24h in all conditions; c) 2-way ANOVA for MCP-1 showing the way effect. *=p<0.05

In b and c, the concentrations at time 0 were subtracted from the medium concentrations at various time points.

In addition to free fatty acids, glycerol, triglycerides and pro-inflammatory markers, we also measured glucose and lactate. Both metabolites were detected with the Yellow Springs Glucose 2300 STAT (Analytical Service srl, Cassina de Pecchi-MI,Italy).

Glucose

Although the experiments were conducted using media with 5.5 mM glucose, representing an initial fasting state, in the 1-way adipose controls glucose levels remained fairly stable over 24 hours, suggesting that a different font of energy was used by the these cells. In the 3-way connection, where the total number of cells was higher, a significant increase of glucose consumption was observed, particularly in the presence of 12%AT and 35%AT. In the 25%AT 3-way configuration glucose uptake was reduced with respect to the other 3-way conditions and medium levels remained stable over 24 hours. The 2-way ANOVA analysis shows significant interaction between the tissue cross-talk and adiposity ($p=0.004$). Glucose uptake in the 1-way connection was almost negligible for all levels of adiposity. As shown in Figure E, glucose uptake in the 3-way system is not correlated with the amount of adipose tissue or the total number of cells but increases significantly in the presence of 12%AT and 35%AT with respect to the 1-way controls ($p<0.005$).

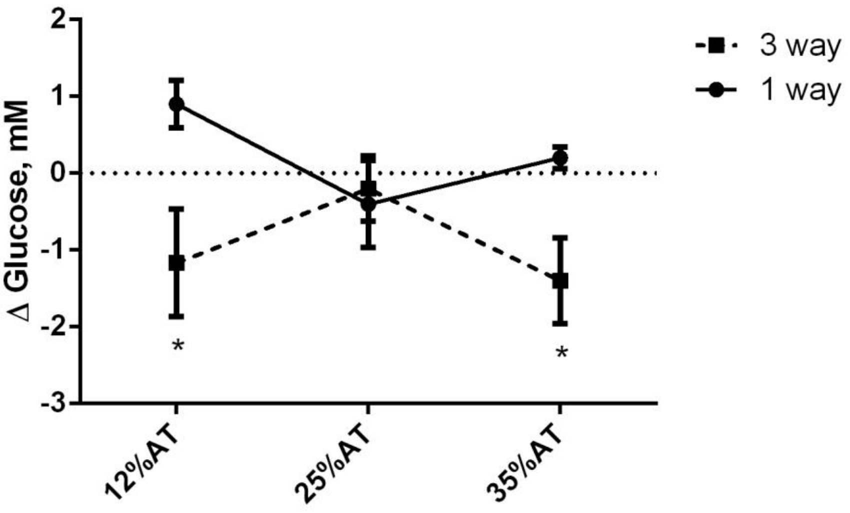

Fig. E. Changes in glucose concentration in control 1-way and 3-way connected cultures as a function of adiposity. * p<0.05 with respect to the corresponding 1-way condition.

Lactate

Lactate is considered one of the main glucogenic precursors in the fasting state (*1*) and adipose tissue is an active producer of this metabolite. In-vivo, lactate production is regulated by the nutritional state and the degree of obesity (*2*). Moreover, increased lactate production is observed in adipocytes derived from obese adipose tissue. A net production of lactate was observed in the 3-way connections (Fig. F, $p<0.0001$ with respect to the 1-way group, ANOVA2), with the interaction plot indicating the synergic effect of inter-tissue cross-talk on lactate production in the 3-way connection compared with the 1-way adiposity controls. In the 1-way control system medium lactate levels were not correlated with the adipose tissue amounts. However, the presence of endothelial cells and hepatocytes in the 3-way circuit determined a significant increase in lactate levels, mirroring the trend observed in-vivo.

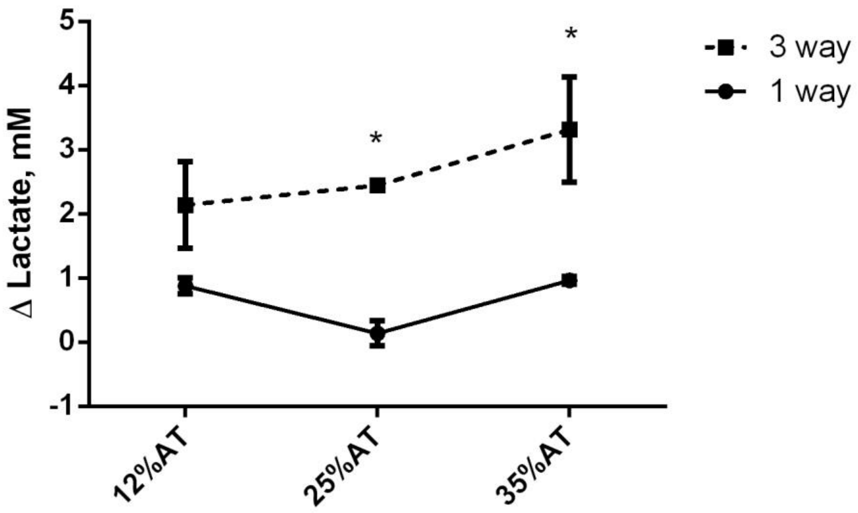

Fig. F. Changes in lactate concentration in control 1-way and 3-way connected cultures as a function of adiposity. Different letters indicate significant differences.

Estimation of Von Willebrand factor (vWF) expression through image processing

The amount of vWF expressed per endothelial cell in different conditions was measured using immunohistochemistry. At the end of the 24 h experiments, the bioreactor circuit was dissembled by first draining the media reservoir and separating the 3 modules. The laminar flow Ibidi slide with HUVEC was treated with 4% paraformaldehyde for 20 mins as per manufacturer's instructions and cells were stained for vWF with Monoclonal Mouse Anti-hvWF clone F8/86 (Dako, Denmark) and anti-mouse FITC (Invitrogen, Paisley, UK) as a secondary antibody; DAPI (4',6-diamidino-2-phenylindole) staining was also performed for nuclear labeling. Fluorescence was visualized using a confocal microscope (Nikon A1). In particular, images were taken using a 10X objective with a pixel-to-micron ratio of 1.23 um/pixel on a 1024x1024 matrix. For the green channel, the same confocal settings were used for all scans (i.e. 25 W laser power). To quantify vWF expression, firstly, the number of nuclei (#nuclei) was calculated on the blue channel of each image using ImageJ. As regards the green channel, a global threshold using the Otsu method (*3*) was performed, then the Mean Pixel Intensity (MPI) per nucleus (MPI/#nuclei) was evaluated. The MPI was calculated using the method described in Gonzalez et al., 2009. (*4*).

$$MPI = \frac{\sum_{i=1}^{i=M} I_M}{M}$$

where M is the number of the object pixels (i.e. the cell regions expressing vWF) and I_M their pixel intensity.

Data were averaged over 3 samples per condition (1-way HUVEC, 1-way HUVEC+10 ng/mL LPS and 3-way 12%AT, 25%AT and 35%AT) and at least 5 regions of interest per sample. Sample images are reported in Figure G.

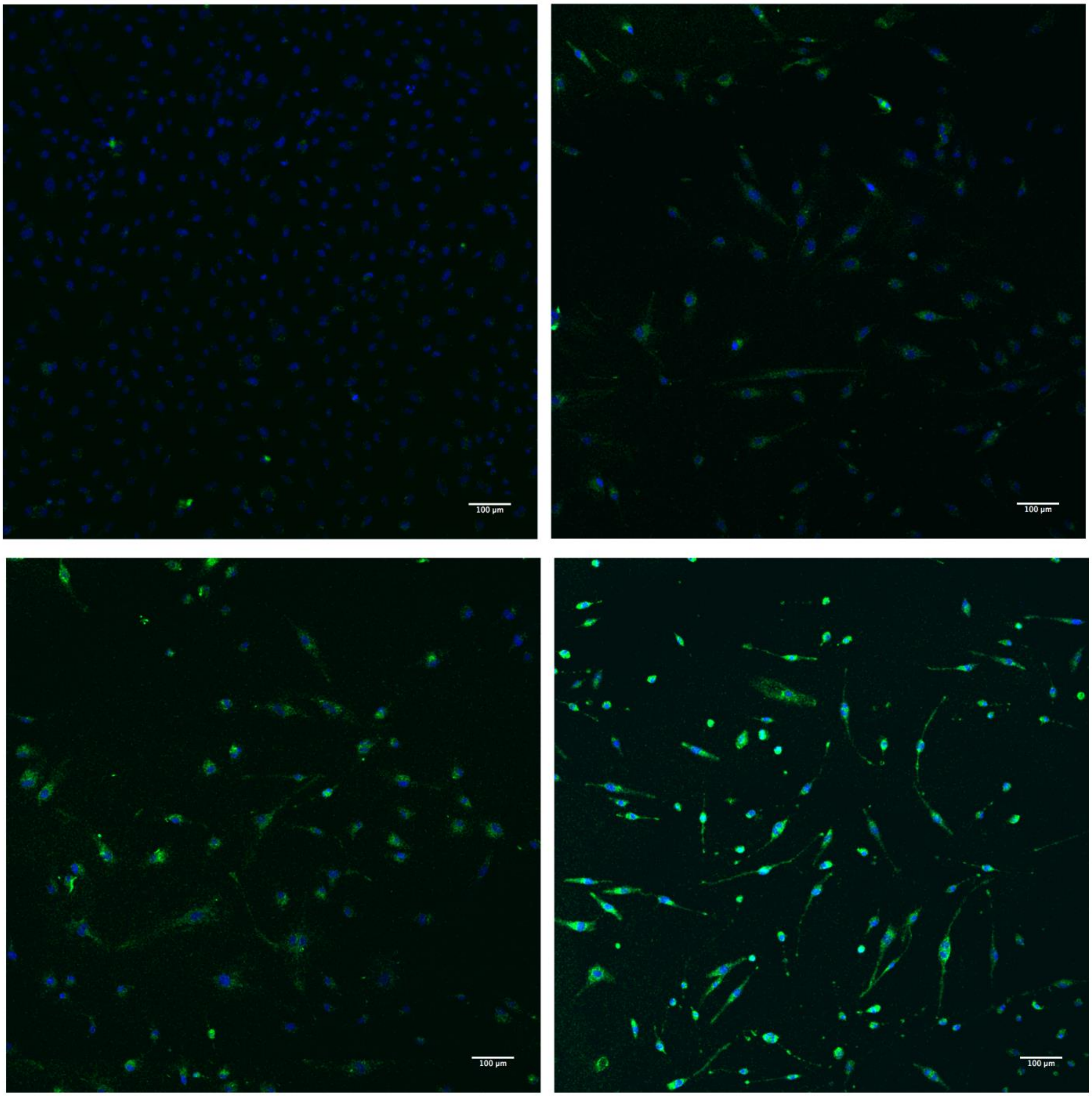

Fig. G. vwF (green), DAPI (blue) stained HUVEC after 24h. A) HUVEC control, B) 3-way 12%AT, C) 3-way 25%AT, D) 3-way 35%AT.

References

1. M. DiGirolamo, F. D. Newby, J. Lovejoy, Lactate production in adipose tissue: a regulated function with extra-adipose implications. *FASEB J.* **6**, 2405–12 (1992).

2. J. Lovejoy, B. Mellen, M. Digirolamo, Lactate generation following glucose ingestion: relation to obesity, carbohydrate tolerance and insulin sensitivity. *Int. J. Obes.* **14**, 843–55 (1990).

3. N. Otsu, A Threshold Selection Method from Gray-Level Histograms. *IEEE Trans. Syst. Man. Cybern.* **9**, 62–66 (1979).

4. R. C. Gonzalez, R. E. Woods, S. L. Eddins, *Digital Image Processing Using MATLAB* (Gatesmark Publishing, Knoxville, TN., 2nd ed.,., 2009; http://www.imageprocessingplace.com/DIPUM-2E/dipum2e_main_page.htm).
